# Supplementary material for: Correlation between higher-order aberration and photophobia after cataract surgery
Source: PLoS One. 2022 Sep 15;17(9):e0274705. doi: 10.1371/journal.pone.0274705 (PMC9477362; doi:10.1371/journal.pone.0274705)
Supplement: S1 Text — (DOCX) [file pone.0274705.s001.docx]

## S1 Text. Photophobia scaling score

Please rate the degree of discomfort with the bright light you experienced under the sun on a scale of zero to ten.

0 - None. No discomfort at all.

1

2

3

4

5 - Moderate discomfort.

6

7

8

9

10 - Severe discomfort. Too severe to open your eyes.
