# Supplementary material for: Correlation between higher-order aberration and photophobia after cataract surgery
Source: PLoS One. 2022 Sep 15;17(9):e0274705. doi: 10.1371/journal.pone.0274705 (PMC9477362; doi:10.1371/journal.pone.0274705)
Supplement: Attachment — Submitted filename: Review for PONE-D-22-10607-wy.docx [file pone.0274705.s002.docx]

Comments:

This manuscript focused on the relationship between the subjective degree of photophobia and HOAs before and after cataract surgery. The purpose of this study was to clarify changes in these two parameters before and after cataract surgery, along with the associations between them. Overall the study was meaningful.

Major issues:

1、 The surface of the cornea is aspheric. However，the central three millimeters of the cornea is nearly spherical and the aberration of three millimeters in the central cornea would not be obvious. What causes the decrease in photophobia changes related with HOA and how it is related to scattering. Please discuss more clearly in Discussion.

2、 There exists gender bias in the study, and there is no analysis of whether the difference is related to gender. It also needs a multivariate model comparing the changes in photophobia and HOAs before and after cataract surgery, adjusting for age, sex at the least…

3、 The definitions of glare and photophobia is similar in this study. According to previous studies, glare is closely related to scattering which is available for clinical measurement. It did not measure it and could not show its relationship with aberration and photophobia in the study. Please briefly analyze in revised text.

Minor issues:

1、 Is the pain scaling score in the study widely used in evaluating photophobia in the ophthalmic literature? Please discuss briefly in revised text. Please provide English version of photophobia scaling score as supplementary files.
