# Supplementary material for: Correlation between higher-order aberration and photophobia after cataract surgery
Source: PLoS One. 2022 Sep 15;17(9):e0274705. doi: 10.1371/journal.pone.0274705 (PMC9477362; doi:10.1371/journal.pone.0274705)
Supplement: Attachment — Submitted filename: Response to Reviewer.docx [file pone.0274705.s003.docx]

# **Response to Reviewer Comments:**

The following pages present the verbatim comments of the reviewer and editor in black**;** our responses to each point are printed in red**.** We highlighted *words* we modified or added in this version of our manuscript. We hope our changes satisfactorily address the comments.

**Reviewer #1: Review of PONE-D-22-10607**

In this well-written paper the authors describe a relationship between photophobia and higher order lens aberrations measured before and after cataract surgery. They show an anticipated reduction in glare following surgery, and a correlation between glare and higher order lens aberrations.

Thank you for your comments and careful review. We answered and revised our manuscript according to your comments one by one.

The authors need to say a little more about how they measured photophobia. “We scored photophobia from 0 (patient does not experience photophobia at all) to 10 (patient experiences severe photophobia to open eyes) … We asked patients to score the degree of photophobia on a sunny day…” There appears to have been little control over the viewing conditions, indeed patients appear to be scoring the photophobia from memory. Please specify exactly how photophobia was measured i.e. the wording of the questions used and the setting in which they were asked.

We agreed with the reviewer's comment. As the reviewer pointed out, the subjective photophobia score was recorded based on the recall method. We are thankful for the reviewer’s comments, which made us aware that we did not write the method clearly in the manuscript. It was only noted in the limitation section of Discussion. Following the comments, we have clarified how we had the scores in the Methods.

**Action**: We revised the methods section about scoring photophobia to document the measurement exactly. In detail, we added sentences in the Methods section as follows;

Revised manuscript page 5, line 111 to 115 in Methods;

*With a subjective photophobia scoring sheet (S1 text), one ophthalmologist asked a patient in a bright exam room, “Please rate the degree of discomfort with the bright light that you experienced under the sun on a scale of zero to ten. A score of zero means that you did not feel discomfort at all, while a score of ten indicates that you felt severe discomfort too much to open your eyes. A score of five means that you felt discomfort moderately."*

The authors acknowledge that large individual differences in self-reported glare occur despite identical “visual inputs”. Some individuals are more susceptible to uncomfortable sensations than others, and this is often related to headache susceptibility. Do the authors have information concerning headaches? Because the susceptibility to uncomfortable sensation varies with individual, the authors are correct to analyse the worst eye, but they refer to “35 of 41 eyes” as if the eyes are independent of the individual, which they are not.

Thank you for the reviewer’s comments. Our clinical study did not include any obvious migraine patients, although our study did not obtain accurate information on headaches. We agreed that we should have gathered and shown the information regarding headaches. So, we added the information that our data include any patient with obvious migraine and we do not have accurate information concerning headache in the limitation section in Discussion. Regrettably, it is currently quite hard to acquire accurate information about headaches because it has been relatively a long time since the data was collected and it is still under the pandemic of COVID-19.

As the reviewer pointed out, we should have described “32 cases” not “32 eyes”. In fact, we performed correlation analysis for only one eye per person. This point has also been corrected in accordance with the reviewer's comments. We are thankful that the reviewer raised this point which could cause readers to misunderstand our paper.

**Action**: We added the information concerning headache which could affect our results in Methods and the limitation section in Discussion. We also changed ‘eye’ into ‘case’.

Revised manuscript page 5, line 106 in Methods;

*Exclusion criteria included patients with any complications until 1 month after surgery, with other co-existing ophthalmic or systemic diseases that affect vision and/or photophobia such as migraine, and who refused to participate. Postoperative data were obtained during 1 month after surgery.*

Revised manuscript page 15, line 375 to 377 in Discussion;

*Further large-scale, longitudinal follow-up studies covering the data of multi-modalities, including threshold measurements for photophobia and information about the degree of ocular pain and headaches, are required to further explore these relationships.*

Revised manuscript page 2, line 34 to 39 in Abstract;

*After cataract surgery, photophobia scores increased, remained unchanged (stable), and decreased in 3, 41, and 27 cases, respectively. In the stable group, 35 of 41 cases belonged to PP unconcerned. In PP concerned, there were significant correlations between photophobia score and postoperative root-mean-square values of total HOAs (rs = 0.52, p = 0.002), total coma (rs = 0.52, p = 0.002), total trefoil (rs = 0.47, p = 0.006), and third-order group (rs = 0.53, p = 0.002).*

Revised manuscript page 8, line 204 to 207 in Results;

*We analyzed the association between subjective photophobia scores and Zernike coefficients. After excluding PP unconcerned patients, which include 3 eyes with inaccurate OPD scan measurements, 32 cases remained as PP concerned cases, that we defined as patients with pre- or postoperative photophobia scores of 0.*

Revised manuscript page 10, line 231 to 232 in Methods;

Fig 3. Correlation between RMS of wavefront errors and photophobia score

*(a) Preoperative and (b) postoperative data are shown. The vertical axis represents positive root-mean-square (RMS) values of wavefront errors. The horizontal axis represents the subjective photophobia score. The distribution of RMS values in 30 cases (PP unconcerned) with a photophobia score of 0 both pre and postoperatively are shown using a violin plot on the left side of each graph. RMS data of other 32 cases (PP concerned) are plotted using red and blue circles.*

The distinction between glare and photophobia needs to be elaborated a little. In particular it would be useful to know whether patients experienced pain. Photophobia often implies discomfort or pain, whereas glare usually implies impaired vision. The distinction is often made in terms of “discomfort glare” and “disability glare”. Were the patients reporting pain (photophobia) or simply glare? I suspect when the scores were low they were reporting disability glare, and when high both disability and discomfort glare. The authors have divided scores into zero and above zero. Is this an appropriate division? Were the high scores associated with headaches?

We greatly appreciate the reviewer's comments. The reviewer's point is related to the 1st and 2nd questions, citing the association between pain, glare, and photophobia. We understand that we should have stated the point more clearly in our manuscript. We added sentences to answer the first question, describing in detail how we asked patients about discomfort with the bright light. Hence, we considered the patients reported discomfort glare rather than disability glare and our current version of manuscript does not mislead readers. However, it is quite hard to distinguish between disability glare and discomfort glare clearly because these two kinds of glare are sometimes intermixed and inseparable. Therefore, we wrote the sentence in Introduction: *“A psychophysical study suggests discomfort glare is closely associated with spatial properties such as simulated glare sources(19),”* We are still interested in the reviewer’s hypothesis, but we will skip to write it in our paper unfortunately because we have no detailed information about headache.

About dealing with PP unconcerned patients (both 0), the reviewer's point is valid. In other words, even if single regression analysis showed no correlation, it is still unclear whether the eliminated data are really meaningless. Therefore, we performed an additional multivariate analysis to validate the concern. In the linear mixed-effects model (LME), the objective variable was the subjective photophobia score, the random effect was the individual patient. The independent variables were calculated as age, gender and RMS of HOAs (total coma and total trefoil). According to the model, postoperative photophobia scores were significantly associated with HOAs (coma and trefoil) in the PP concerned group (excluding 0 to 0) as in the results of the single regression. Also, subjective PP scores in females were higher than males. In contrast, preoperative PP scores were not significantly associated with all variables. These results suggest that the subjective photophobia score in the PP unconcerned group was presumably not meaningful and the data could have been properly understood by analyzing them separately in this study. We thank the reviewer’s comments for this opportunity to draw a more careful conclusion.

**Action**: We added sentences to describe an additional multivariate analysis in Methods and Results.

There are a few minor infelicities of English usage as follows:

“impinged spatial properties and wavelength distribution on retinal images.”

“decreased in postoperatively”

“alternation in photophobia” instead of alteration in photophobia

Thank you for your corrections.

Action: We fixed a few minor infelicities of English usage and typo.

**Reviewer #2:**

The manuscript describes a technically reliable scientific study with data supporting the conclusion. The study was carried out strictly with proper control, replication and sample size. This study draws appropriate conclusions based on the data provided.

We appreciate you for reviewing our study.

**Reviewer #3 (from an attached word file) :**

This manuscript focused on the relationship between the subjective degree of photophobia and HOAs before and after cataract surgery. The purpose of this study was to clarify changes in these two parameters before and after cataract surgery, along with the associations between them. Overall the study was meaningful.

Thank you very much for your review and meaningful comments.

Major issues:

1. The surface of the cornea is aspheric. However, the central three millimeters of the cornea is nearly spherical and the aberration of three millimeters in the central cornea would not be obvious. What causes the decrease in photophobia changes related with HOA and how it is related to scattering. Please discuss more clearly in Discussion.

We thank the reviewer for raising this very fundamental and important point. We consider that the main causes of photophobia are different preoperatively and postoperatively. That is, as previously reported, preoperative photophobia is strongly influenced by the forward light scatter from the cataract, masking the relatively weak effect of higher-order aberrations. Forward scattering and aberrations caused by cataracts are removed by cataract surgery. So after surgery, the higher order aberrations decrease. We consider that the residual postoperative higher order aberrations of the whole eye, which are thought to arise primarily from the cornea, are one factor that influences postoperative photophobia.

Furthermore, we estimated the point spread function with total higher-order aberrations in representative patients. There appears to be no obvious difference in PSF between the aberration-free ideal observer (the left column) and all subjects at 550 nm. However, as the wavelength gets shorter, the PSFs of two of the left columns (the ideal observer and the subject with small aberrations) and two of the right columns (those of the two subjects with large higher-order aberrations and high subjective PP score) differ greatly. The difference in PSF of HOAs of the whole eye at shorter wavelengths might be related to subjective photophobia scores.

Action: Following the reviewer’s comments, we added sentences to discuss scattering and HOAs more clearly. Also, we added Figure 4 to show the PSFs in representative subjects in Discussion.

Revised manuscript Page 14 line 320 to 325 in Discussion;

*We calculated the PSFs from HOAs in the ideal observer (no HOAs) and representative patients (Fig 4). There was no obvious difference in PSF between the aberration-free ideal observer (the left column) and all participants at 550 nm. However, as the wavelength was shorter (500nm), the PSFs of two of the left columns (the ideal observer and the subject with small aberrations) and two of the right columns (those of the two participants with large higher-order aberrations and high subjective PP score) differ greatly.*

Revised manuscript Page 14 line 330 to 333 in Discussion;

*Additionally, alteration of the yellowish crystalline lens to clear intraocular lens by cataract surgery increases shorter wavelength light which facilitates photophobia. That PSFs at shorter wavelengths were more affected with HOAs, as shown here, may also increase subjective photophobia scores.*

2. There exists gender bias in the study, and there is no analysis of whether the difference is related to gender. It also needs a multivariate model comparing the changes in photophobia and HOAs before and after cataract surgery, adjusting for age, sex at the least…

We are greatly thankful of the reviewer’s comment to improve our manuscript. Following the comments,　we performed a multivariate analysis using RMS of HOAs (total coma and total trefoil), gender and age because pain and light sensitivity is associated both with gender and age in previous studies as the reviewer mentioned. We used a linear mixed-effects model for a total 2 x 3 which is six sets of data: two sets of data for all patients group and the PP concerned patients group, and three sets of data for preoperative only, postoperative only, both pre- and postoperative. Similar to the results of the single regression analysis, we found that RMS of total coma and total trefoil were associated with subjective photophobia scores in the postoperative PP concerned group. In addition, females were associated with photophobia score, but not age. In the other data sets, the results were not or weakly associated with photophobia score. The results of the multivariate analysis were added to Results, and gender differences for photophobia were discussed in Discussion.

Action: Now we have new sections about multivariate analysis in Methods, Results and Discussion.

Revised manuscript Page 6 line 141 to 147 in Methods;

*Furthermore, to address the possibility of misunderstanding our data with a single linear regression analysis alone, a linear mixed model was used for a total of 2 x 3, which is six sets of datal; two sets of data for all patients and PP concerned patients, and three sets of data for preoperative, postoperative and both. In the model, subjective photophobia score was the response, RMS of each HOA group (total coma (µm) and total trefoil), gender and age were fixed effects and individuals were the random effect because light sensitivity is associated both with gender[35] and age [36]. A p-value of <0.05 was considered statistically significant in multivariate analysis.*

Revised manuscript Page 11 line 248 to 261 in Results;

*Association between photophobia scores and HOAs or gender*

*The relationship between subjective photophobia score and RMS of HOAs (total coma and trefoil), age or gender was analyzed in a linear mixed-effect model. These results are shown in Table 4. According to this model, subjective postoperative photophobia score in PP concerned patients was positively associated with RMS of a total coma of 34.27 µm (95% CI, 10.42 – 58.13 µm; p < 0.01) and RMS of total trefoil of 13.4 µm (95% CI, 1.52 - 25.32 µm; p < 0.05). Subjective photophobia scores in females were higher than in males in three data sets; postoperative PP concerned patient, all postoperative patients and all pre- and postoperative all patients. In contrast, preoperative photophobia scores in all patients and PP concerned patients were not associated with all of 4 variables.*

Revised manuscript Page 15 line 346 to 362 in Discussion;

*Non-visual factors and subjective photophobia score*

*We have discussed photophobia induced by visual input. However, other neural inputs, namely trigeminal inputs, have long been considered to contributed to photophobia[23]. Anterior eye diseases such as keratitis and iritis can cause photophobia[22]. In addition, there are several reports of photophobia enhanced by migraine[62-64] associated with trigeminal nerve input. In rodents, cells that receive trigeminal and visual inputs were found in the posterior thalamus[65]. Furthermore, the association between inputs to the ipRGC with a strong response to short-wavelength light and photophobia during migraine has been reported in humans[34]. Thus, pain and photophobia are considered strongly related sensations. Gender is an important factor in pain sensitivity differences[66]. Females are 2-3 times more likely than males to have migraines due to hormonal differences[67] while no gender differences exists in photophobia in visually normal people[36], and males have higher brightness perception than discomfort[35]. In our results, females had significantly higher subjective photophobia scores than males, suggesting the influence of postoperative subthreshold trigeminal inputs that a patient never perceive because females demonstrate heightened central sensitization[68]. Visual photosensitivity threshold was reported to increases with age in visually normal subjects[36]. Although the effect of age was not observed in photophobia scores in our data, this might be because patients underwent surgery for visual impairment due to cataracts; thus were older and had a relatively narrower range of age.*

3. The definitions of glare and photophobia is similar in this study. According to previous studies, glare is closely related to scattering which is available for clinical measurement. It did not measure it and could not show its relationship with aberration and photophobia in the study. Please briefly analyze in revised text.

We appreciate the reviewer’s comment and agree that our study uses a similar definition of "disability" glare and photophobia, and that "disability" glare is closely related to clinically measurable forward scattering. However, forward scatter was not measured due to lack of measurement equipment unfortunately. Previous studies have reported that aberration does not correlate with forward scatter. In this study, both in the single regression and in the results of the additional multivariate analysis, the effect of aberration on photophobia scores was found in the postoperative data and could not be explained by the preoperative data. Our results were not able to certify directly the influence of forward scattering, but implicitly supports the influence of preoperative scattered light because preoperative photophobia score presumably requires other variables that we did not measure in our study. We discuss the points in the Discussion.

Actions: Following the reviewer’s comments, we modified the sentences about forward scattering in Discussion.

Revised manuscript Page 14 line 335 to 336 in Discussion;

*Notably, our results show that no component of preoperative HOAs was significantly associated with photophobia score and could not be explained by the previous interpretation using retinal inputs and HOAs, although we analyzed with not only single linear regression but also multivariate analysis. We considered the results implicitly supporting the influence of another factor not measured - forward scattering. Thus, we interpreted the photophobia induced by forward scattered light preoperatively masked the photophobia induced by HOAs, which may be relatively small, in cataract.*

Minor issues:

1、 Is the pain scaling score in the study widely used in evaluating photophobia in the ophthalmic literature? Please discuss briefly in revised text. Please provide English version of photophobia scaling score as supplementary files.

We agreed with the reviewer’s comments. Although there are no standardized methods in an ophthalmology clinic to assess photophobia, qualitative evaluation or scaling is often used. In addition, we provided an English version of the photophobic scaling score in this study.

Action: We added sentences and citations about scaling score in Methods. We provided the photophobia scaling score as S1 text.

Revised manuscript Page xx line xx

*Although no standardized method has been established to measure the degree of photophobia quantitatively,* we scored photophobia from 0 (patient does not experience photophobia at all) to 10 (patient experiences severe photophobia to open eyes) with reference to the pain scaling score, and interviewed patients before and after cataract surgery as in a previous study*[32-34]*.

*S1 Text. Photophobia scaling score.*

*Please rate the degree of discomfort with the bright light that you experienced under the sun on a scale of zero to ten.*

*0 - None. No discomfort at all.*

*1*

*2*

*3*

*4*

*5 - Moderate discomfort.*

*6*

*7*

*8*

*9*

*10 - Severe discomfort. Too severe to open your eyes.*
